# Drug-Resistant Tuberculosis in Prisons of Latin America and the Caribbean: A Critical Reflection on Structural Challenges and Gaps

**DOI:** 10.3390/tropicalmed11040088

**Published:** 2026-03-24

**Authors:** Ariel Torres, Gisselle Trujillo, José Daniel Sánchez

**Affiliations:** Facultad de Ciencias de la Salud y Bienestar Humano, Universidad Tecnológica Indoamérica, Quito 170103, Ecuador; arieltorres@uti.edu.ec (A.T.); gisselletrujillo@uti.edu.ec (G.T.)

**Keywords:** Latin America, prisons, prison health, MDR-TB, drug-resistant tuberculosis

## Abstract

Drug-resistant tuberculosis (DR-TB) represents a major public health threat, particularly in the prisons of Latin America and the Caribbean, where rates are up to 40 times higher than those observed in the general population. These facilities act as community amplifiers due to overcrowding, poor ventilation, diagnostic delays, and treatment discontinuity. This study offers a critical reflection on the magnitude, determinants, and implications of DR-TB in regional penitentiary contexts. A reflective analytical review was conducted in PubMed, Scopus, Web of Science, SciELO, and LILACS, complemented by WHO and PAHO reports, prioritising studies from 2019 to 2024. The findings reveal MDR-TB and pre-extensively drug-resistant (pre-XDR) outbreaks in Peru, Paraguay, and the Dominican Republic, as well as community transmission linked to prisons in Brazil and Colombia. Persistent gaps remain in systematic screening, drug susceptibility testing coverage, and post-release follow-up. Scientific production continues to be uneven and predominantly biomedical, with limited consideration of social and human rights determinants. DR-TB in prisons reflects the structural deficiencies of health and justice systems; its control requires intersectoral policies, genomic surveillance, and strategies that ensure early diagnosis, treatment continuity, and dignified detention conditions.

## 1. Introduction

Globally, drug-resistant tuberculosis (DR-TB) remains a significant threat, with a particularly severe impact on vulnerable populations such as people deprived of liberty [1,2]. In the late 1980s and early 1990s, a pioneering study in Latin America revealed the extent of initial drug resistance in tuberculosis cases: one in six untreated patients already presented resistance to at least one antituberculosis drug [3]. The most affected drugs were isoniazid and streptomycin, alone or combined, whilst localised foci of rifampicin resistance were detected, highlighting the urgency of strengthening epidemiological surveillance and control strategies against resistant TB [3].

In recent decades, the situation has become even more critical within prison contexts [4,5]. Although tuberculosis incidence has declined in much of the world, in Central and South America it has remained stable or even increased [6,7]. Between 2000 and 2018, the incarcerated population in the region rose by 206%, from 493,000 to 1.35 million people, whilst TB cases reported among people deprived of liberty increased by 269% [6]. Today, although they represent less than 1% of the total population, people deprived of liberty account for nearly 11% of tuberculosis cases in Central and South America [6].

The structural conditions of penitentiary centres, characterised by overcrowding—with an average occupancy rate of 167% and extreme figures such as those in El Salvador (348%)—create an ideal environment for the transmission and persistence of tuberculosis [8,9]. In several countries of the region, including Venezuela, Paraguay, Ecuador, El Salvador and Guatemala, prison TB rates exceed those of the general population by up to 40 times [6,7]. Furthermore, the population attributable fraction for incarceration almost doubled between 2011 and 2017, from 4.5% to 9.7%, representing over 16,000 additional cases in the most recent year recorded [6]. This increase even surpassed the impact of classical risk factors such as diabetes, smoking, alcohol consumption, or HIV co-infection [4,6].

This scenario shows that although drug-resistant tuberculosis is a global problem, it takes on a particularly critical dimension in penitentiary settings, where biological, social, and structural vulnerabilities converge. The reduction in cases in the general population contrasts with the sustained increase in prisons, which have become true epicentres of transmission. Addressing resistant TB in prisons is therefore an essential health and social priority to protect both people deprived of liberty and the community at large. Accordingly, the present study aimed to carry out a descriptive-analytical reflection to critically examine the magnitude, determinants, and structural challenges of drug-resistant tuberculosis in prisons across Latin America and the Caribbean.

## 2. Materials and Methods

This study is framed within a reflective review approach, a methodology that combines scientific evidence with a critical and comparative analysis aimed at deepening conceptual understanding rather than achieving exhaustive quantitative coverage [10]. This type of review is particularly valuable for exploring complex public health problems in contexts where research is fragmented or limited, providing an interpretative perspective that can guide the design of policies and strategies [11,12]. Unlike narrative or scoping reviews, this reflective review does not aim at exhaustive mapping of the literature but focuses on critical and comparative interpretation of heterogeneous evidence. Within this framework, the issue of drug-resistant tuberculosis in prisons across Latin America and the Caribbean was addressed, analysing its magnitude, determinants, and structural challenges.

The search, selection, and analysis of information were conducted between June and August 2024 in the main biomedical databases (PubMed/MEDLINE, Scopus, Web of Science, SciELO, and LILACS), complemented by institutional documents from the World Health Organization (WHO) and the Pan American Health Organization (PAHO), in order to integrate scientific evidence with a regional public health perspective. A total of 165 records were initially identified through searches conducted in these databases and institutional repositories. After duplicate removal (*n* = 28), 137 records underwent title and abstract screening, of which 69 were excluded as they did not specifically address drug-resistant tuberculosis in prison settings in Latin America and the Caribbean. The remaining 68 full-text articles and reports were assessed for eligibility, with 27 excluded due to insufficient data on the prison population or lack of relevance to drug resistance. Finally, 41 sources (peer-reviewed articles and institutional reports) informed the final reflective analysis. Publications from 2019 to 2024 were prioritised in English and Spanish. As this study was conceived as a reflective analytical review rather than a systematic review, it was not designed to strictly follow PRISMA guidelines. Instead, sources were selected based on their relevance and contribution to a critical and comparative interpretation of heterogeneous evidence on drug-resistant tuberculosis in prison settings in Latin America and the Caribbean. The full database-specific search strings are provided in Supplementary Material S1. In addition, the reference lists of included sources were manually screened to identify further relevant publications.

Combinations of descriptors in English and Spanish were used, including: “drug-resistant tuberculosis”, “multidrug-resistant tuberculosis”, “MDR-TB”, “prisons”, “prison”, “Latin America”, and “carceral settings”. Eligible sources were limited to publications in English and Spanish, as these represent the main languages of scientific production and policy documentation relevant to Latin America and the Caribbean and were the working languages of the research team.

The inclusion period focused primarily on publications from the past five years (2019–2024), ensuring both the timeliness and relevance of the evidence. Additionally, several earlier studies of historical or pioneering nature were included for their contextual value in illustrating the evolution of drug resistance in the region. Inclusion criteria encompassed original articles, reviews, meta-analyses, technical reports, and guidelines addressing drug-resistant tuberculosis (MDR/XDR-TB) in Latin American prison populations, contributing epidemiological, clinical, social, or programmatic information. Duplicate studies, opinion pieces lacking empirical support, and reports that did not specify data related to prison contexts were excluded.

No formal quality appraisal or risk-of-bias tool was applied, as the included sources were heterogeneous in design (peer-reviewed studies, reviews, and institutional reports) and the objective of this reflective analytical review was critical interpretation rather than quantitative synthesis. Although an informal assessment of credibility and relevance was conducted during the selection process, the absence of systematic quality evaluation should be considered a methodological limitation. Therefore, the findings should be interpreted with caution, particularly when comparing estimates across countries and data sources.

The information was organised regionally to reflect the geographic and epidemiological heterogeneity of the phenomenon. Three main analytical blocks were established:

- Southern Cone: Brazil, Paraguay, Chile, Argentina, and Uruguay.
- Andean Region: Peru, Colombia, and Ecuador.
- Caribbean: Cuba, Haiti, and the Dominican Republic.

Within each region, three fundamental dimensions were analysed: the epidemiological situation of drug-resistant tuberculosis in prisons, the structural and social factors promoting its spread, and the main challenges and proposals for its control. The analysis was carried out from a reflective and critical perspective. Rather than merely summarising findings, the information was contrasted across countries and regions, seeking to identify shared patterns, knowledge gaps, methodological inconsistencies, and relevant insights. This interpretative process allowed for the integration of biomedical evidence with social and structural determinants, providing a more comprehensive understanding of the issue. From this, the discussion was oriented towards practical implications for public health, clinical care, and future lines of research.

**Figure 1:**
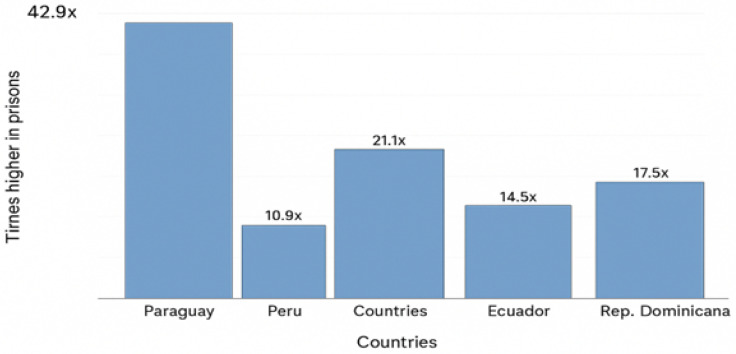
MDR-TB rate ratio: prison population vs. general population in selected Latin American countries. The figure shows illustrative estimates that should be interpreted with caution due to heterogeneity in surveillance systems and diagnostic strategies across countries. Paraguay shows the highest disparity (42.9×), followed by Brazil (21.1×), Dominican Republic (17.5×), Ecuador (14.5×), and Peru (10.9×). These ratios are indicative of the relative magnitude of risk in prison settings compared to the general population within each country.

To complement the descriptive analysis, the rate ratio of multidrug-resistant tuberculosis (MDR-TB) between the prison population and the general population was estimated for countries with available data. The calculation was performed by dividing the MDR-TB rate in prisons (cases per 100,000 persons deprived of liberty) by the corresponding rate in the general population of the same country and period, thus expressing how many times higher the incidence in prisons was compared with that of the community, evidencing the relative magnitude of the risk. Prison and population rates were obtained from official epidemiological sources and recent publications, prioritising regional comparability. Based on these data, Figure 1 was constructed, representing the estimated ratios for five selected countries (Paraguay, Peru, Brazil, Ecuador, and the Dominican Republic).

The main limitations of this review include the methodological heterogeneity of the sources and the reliance on secondary data. As much of the information derives from studies with varying approaches (ecological, retrospective, or institutional), the actual burden of drug-resistant tuberculosis in prison contexts may be underestimated. The absence of systematic quality assessment is an additional limitation. These limitations are inherent to reflective studies, whose purpose is not quantitative exhaustiveness but the critical and comparative integration of the available evidence.

## 3. Synthesis of Evidence

### 3.1. Southern Cone

**Brazil:** In the state of Paraná, an ecological study conducted between 2008 and 2018 revealed a sustained increase in drug-resistant tuberculosis (DR-TB) among people deprived of liberty, with marked geographic concentrations in certain municipalities [13]. This finding indicates that prisons act not only as reservoirs of the disease but also as territorial hotspots of high transmission, reinforcing the need to apply timely molecular diagnostics and ensure continuity of treatment. Complementarily, another analysis carried out in the same region showed that, among MDR-TB cases in the prison population, factors such as previous treatment history, age, and the presence of comorbidities were key determinants in the acquisition and spread of resistance [14]. In addition, intraprison transmission chains were identified, confirming the role of prisons as settings where multidrug-resistant tuberculosis persists and amplifies [14].

**Paraguay:** The magnitude of the problem in Paraguay is equally alarming. A cohort of nearly 3000 people deprived of liberty reported a tuberculosis incidence exceeding 3000 cases per 100,000 inhabitants—extraordinarily high figures compared to the general population [9]. Even more concerning is that the risk of developing the disease persists several years after release [15], underscoring the urgency of strengthening prevention and follow-up measures both within prisons and after individuals regain freedom. Furthermore, a genomic surveillance study conducted in Asunción and Ciudad del Este analysing 471 isolates identified frequent recent transmission within penitentiary centres and direct connections with nearby communities. This finding confirms that prisons act as true “community amplifiers”, extending risk beyond their walls and highlighting the need for integrated policies linking prison and community health systems [7].

**Chile:** Significant gaps persist between the prison population and the general population regarding tuberculosis incidence and treatment outcomes. In 2022, the rate among people deprived of liberty reached 146.3 per 100,000 inhabitants—a 53% increase compared with the previous year [16]. Although this group represented only 2.5% of national cases, the accumulation of risk factors in prisons is evident [17]. Moreover, rifampicin resistance rose from 1.0% in 2014 to 2.2% in 2019, in parallel with the adoption of shorter oral regimens [18]. These data highlight the urgency of strengthening active case finding, expanding molecular testing, and ensuring effective treatments in penitentiary settings.

**Argentina:** National reports show a TB rate in prisons of approximately 319 per 100,000 inhabitants, more than ten times higher than in the general population. However, significant limitations persist: between 2019 and 2021, only 33% of cases underwent drug susceptibility testing, and eight MDR-TB cases were documented. People deprived of liberty constitute a priority group in surveillance policies, accounting for 28.8% of all sensitivity tests recorded. These figures reflect not only the high disease burden but also gaps in timely diagnosis and therapeutic monitoring, underscoring the need to expand DST coverage and reinforce DR-TB surveillance in this vulnerable group [19,20].

**Uruguay:** Recent studies have offered an innovative perspective on tuberculosis in prison settings. A study published in *Microorganisms* identified a specific *Mycobacterium tuberculosis* lineage circulating exclusively within prisons and not detected in the community. Although no high-confidence resistance mutations were found, the results confirmed active transmission within prisons and highlighted the value of applying low-cost sequencing techniques to strengthen epidemiological surveillance [21]. Additionally, the 2024 national report documented 137 tuberculosis cases in penitentiary facilities, representing 11% of the national total, with an incidence of 849 per 100,000 people deprived of liberty and 40% of diagnoses made at the time of entry [22]. These findings emphasise the importance of systematic screening upon entry and continuous monitoring during incarceration as key strategies to interrupt transmission and promote early detection of resistant TB.

### 3.2. Andean Region

**Peru:** Evidence from Peru depicts a particularly concerning scenario. In Callao, genomic analyses identified MDR and pre-extensively drug-resistant (pre-XDR) TB clusters—defined as MDR-TB with additional resistance to either a fluoroquinolone or a second-line injectable agent [23]—dominated by the LAM 4.3.3 strain, as well as a strong association between imprisonment history and membership in recent transmission chains [24]. Nationally, a large-scale screening programme including over 38,000 incarcerated individuals demonstrated the effectiveness of combining chest X-ray screening with rapid molecular tests, allowing detection of tuberculosis and rifampicin-resistant TB even in asymptomatic individuals [25]. Similarly, in juvenile centres, a considerable hidden burden was observed, with 42.8% rifampicin resistance among confirmed cases—revealing an emerging risk in young populations [26]. Together, these findings indicate that Peru faces an expanding epidemic of DR-TB in its prison system, and that integrating advanced technological tools with active case-finding strategies must become an essential component of the national tuberculosis response.

**Colombia:** Studies conducted in prisons in Bucaramanga and Medellín confirmed the presence of recent transmission clusters of resistant tuberculosis, evidencing the active circulation of the disease within prisons and the need to strengthen both molecular diagnosis and contact control actions [27]. At the national level, an analysis of 1372 MDR/RR-TB patients between 2009 and 2020 showed a progressive increase in cases, concentrated mainly among middle-aged men in urban areas with greater diagnostic capacity [5]. Indigenous populations, prisoners, and homeless individuals exhibited markedly higher rates than the general population, whereas no significant differences were observed among Afro-Colombians. Most cases corresponded to pulmonary forms (92%), whilst extrapulmonary forms were linked to better diagnostic access. In terms of pharmacological profile, high sensitivity (>97%) was observed for levofloxacin, capreomycin, amikacin, and moxifloxacin; however, notable resistance rates were reported for ethionamide (29%), pyrazinamide (43%), and ethambutol (25%), presenting major therapeutic challenges [5]. Overall, MDR/RR-TB continues to rise, concentrated among the most vulnerable groups, calling for strengthened molecular surveillance and optimised treatment regimens.

**Ecuador:** A cross-sectional analysis in national prisons revealed that the TB burden among people deprived of liberty is closely linked to social and institutional determinants such as unequal access to timely diagnosis and lack of continuity of care. These limitations create conditions conducive to the progression and transmission of resistant TB [28]. Evidence suggests that Ecuador’s structural prison system gaps hinder both detection and proper management of cases, highlighting the need to prioritise systematic screening and drug susceptibility testing (DST) to strengthen the national response [29]. Likewise, a study conducted in a Guayaquil prison (Centro de Rehabilitación Social Varones No. 1) analysed 36 clinical isolates using MIRU-VNTR typing (15 loci). A high prevalence of the Euro-American lineage, particularly the LAM sublineage, was observed, along with MDR-TB cases, underscoring the urgent need to improve diagnostic, treatment, and epidemiological and genotypic surveillance strategies in prison settings [30].

### 3.3. Caribbean

**Cuba:** Reports indicate that TB incidence in prisons is up to eight times higher than in the general population, with outbreaks driven by overcrowding and diagnostic delays. Between 2020 and 2022, MDR-TB cases were reported among prisoners. A retrospective multicentre study determined that around 2% of confirmed cases corresponded to MDR-TB, although evident underreporting in the penitentiary system was noted—highlighting the need to implement systematic molecular testing to improve detection and control in this high-risk group [31]. Between 2015 and 2017, Cuban isolates of *Mycobacterium tuberculosis* from pulmonary TB patients were evaluated for drug resistance. 93.2% were sensitive to isoniazid and rifampicin, whilst 39 cases showed resistance to isoniazid, 23 to rifampicin, and 10 with multidrug resistance [32]. No resistance to second-line drugs was detected. These results reveal a growing trend of resistant tuberculosis and reinforce the importance of investigating its causes and strengthening prevention, diagnostic, and control strategies in the country [33].

**Haiti:** The prison situation reflects profound vulnerability to tuberculosis. In Port-au-Prince, an outbreak with more than 50 confirmed TB cases was reported, of which 18 were multidrug-resistant (MDR), in a context of extreme overcrowding exceeding 300% capacity. The response included isolation measures, molecular sensitivity testing, and oral therapeutic regimens adapted to available resources [34]. Nationally, it is estimated that about 12% of the total TB burden occurs among prisoners, with a 5.7% prevalence of MDR-TB in isolates from these settings. Factors such as political instability, limited resource availability, and the absence of systematic screening programmes constitute major barriers to effective disease control and perpetuate transmission [35].

**Dominican Republic:** Multidrug-resistant tuberculosis (MDR-TB) has historically been one of the country’s major public health challenges, identified by WHO in the 1990s as a global hotspot. Although international guidelines have been adopted and specific projects implemented for its control, limitations persist that reduce the effectiveness of interventions. The National Survey of Catastrophic Costs of Tuberculosis (ECCTB-RD) found that 8% of patients had MDR-TB and that factors such as low educational level, informal employment, and HIV co-infection increased the risk of facing catastrophic costs associated with the disease [36]. In 2022, 4306 TB cases of all forms were reported (40.5 per 100,000 inhabitants), representing a 6.4% increase compared to 2021, attributed to expanded diagnostic coverage and active case-finding in vulnerable populations [36]. These data reflect both the progress achieved and the ongoing challenges to achieving effective control of resistant tuberculosis in the country.

### 3.4. Illustrative Analysis of Epidemiological Disparity

The following section presents an illustrative comparison of the magnitude of multidrug-resistant tuberculosis (MDR-TB) in prison populations versus the general population in selected Latin American countries. Figure 1 shows the prison-to-community MDR-TB rate ratio, highlighting substantial differences between these settings. The highest ratio was observed in Paraguay (42.9 times higher), followed by Brazil (21.1), the Dominican Republic (17.5), Ecuador (14.5), and Peru (10.9). These findings should be interpreted with caution, as cross-country comparisons may be influenced by differences in surveillance systems, diagnostic capacity, and reporting practices. The observed differences are illustrative and indicative rather than directly comparable estimates or causal conclusions. Nevertheless, the results underscore that prisons can represent high-risk environments for MDR-TB transmission and reinforce the need to strengthen timely diagnosis and continuity of care in these settings.

### 3.5. Synthesis of Problems and Gaps

A structured synthesis of the main problems and gaps identified in research on drug-resistant tuberculosis (DR-TB) in penitentiary contexts across Latin America and the Caribbean is presented below. Table 1 aims to provide an overview of the limitations identified in the literature, avoiding redundancy within the main text. Its purpose is to highlight critical aspects that shape current knowledge and, at the same time, guide future priorities for research, cooperation, and public policy formulation in the region.

## 4. Discussion

The review of evidence on drug-resistant tuberculosis (DR-TB) in penitentiary centres across Latin America and the Caribbean reveals a complex scenario, where the disease is closely linked to structural, social, and programmatic determinants. Although progress has been made such as the implementation of genomic surveillance and large-scale screening in Peru and Paraguay [9,24], research remains fragmented and methodologically heterogeneous, hindering regional comparisons and the development of evidence-based policies.

One of the most significant issues identified (Table 2) is the unequal scientific production. Brazil, Peru, Paraguay, and Colombia concentrate the most consistent studies, whilst other countries show a marked scarcity of data [6,13]. This imbalance prevents a full understanding of the regional magnitude of the problem and weakens international cooperation efforts.

**Figure 2:**
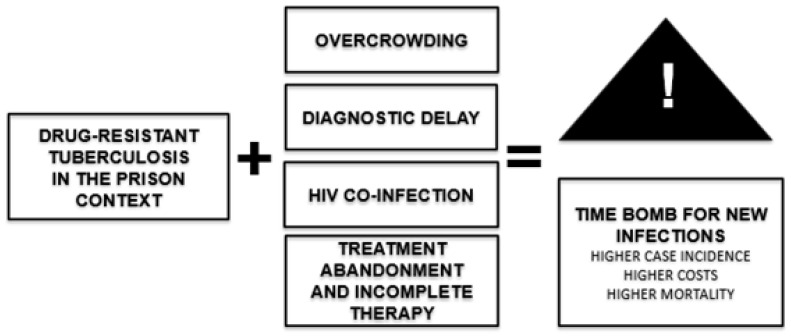
Critical factors that exacerbate drug-resistant tuberculosis in prison settings and their epidemiological consequences. Source: Author’s own elaboration.

Overcrowding and the structural deficiencies of prison systems create a conducive environment for the transmission of *Mycobacterium tuberculosis*. In several countries, TB rates in prisons exceed those in the general population by up to 40 times, as observed in Paraguay and El Salvador [6,7]. Nevertheless, institutional responses remain focused on the biomedical component—diagnosis and treatment—without addressing in a structural manner a reduction in overcrowding or the continuity of care after release (Figure 2).

In addition, there is weak coordination between prison systems and national tuberculosis control programmes. Although some countries have incorporated rapid molecular testing and new therapeutic regimens, coverage remains uneven, with diagnostic delays and limited implementation of drug susceptibility testing [5,16]. The lack of therapeutic continuity after release constitutes a blind spot that facilitates the community spread of resistant strains.

Beyond diagnosis and treatment availability, systematic factors that drive the *de novo* development of drug resistance require urgent attention. Reliable administration through directly observed therapy (DOT), adequate and uninterrupted drug supply, and proper adherence support are critical to preventing the emergence of resistance [4,23]. In prison settings, these elements are frequently compromised due to resource constraints, staff shortages, and institutional instability. Strengthening DOT implementation, ensuring consistent availability of quality-assured medications, and establishing robust supply chain systems are essential components of a comprehensive DR-TB control strategy in correctional facilities.

Another critical element is the limited integration of social determinants and human rights in the analysis of MDR-TB. Factors such as poverty, informal employment, low educational level, and HIV co-infection influence the disease burden and the catastrophic costs of treatment [28,36]. However, few studies address these comprehensively, limiting the development of sustainable, person-centred policies.

In this context, a multidisciplinary and intersectoral approach that transcends the biomedical perspective is imperative. It is essential to strengthen research, epidemiological surveillance, and cooperation among health, justice, and human rights systems. Prisons must be understood as true “community amplifiers” of drug-resistant tuberculosis [7,17].

In summary, controlling MDR-TB in Latin American prisons requires overcoming three core challenges:

1. The scarcity of systematic evidence.
2. Institutional fragmentation.
3. The lack of attention to structural determinants.

Only through coordinated regional scientific production, effective integration between health and justice programmes, and humane, sustainable prison policies will it be possible to contain the spread of resistant TB and mitigate its impact on both people deprived of liberty and the wider community.

This situation carries implications for clinical practice, public health, and research.

**Clinical practice:**

- The lack of rapid and systematic drug susceptibility testing leads to delayed diagnoses, inadequate treatments, and an increased risk of XDR-TB.
- The discontinuity of treatment after the release of people deprived of liberty favours relapses and community transmission.

**Public health:**

- Overcrowding and poor infrastructure turn prisons into hotspots for the amplification of resistant TB.
- Weak coordination between prison and national programmes perpetuates diagnostic and therapeutic gaps.
- The neglect of social determinants and HIV co-infection limits the effectiveness of interventions.

**Research:**

- The scarcity of studies prevents accurate estimation of the true burden of MDR-TB in prisons and leads to policies based on incomplete information.
- Methodological heterogeneity limits regional comparability and the detection of transmission patterns.
- The limited application of genomic surveillance restricts understanding of predominant lineages.

Drug-resistant TB in penitentiary contexts is not merely a health problem among marginalised populations but a reflection of the structural weaknesses of health and prison systems. Prisons continue to function as “community amplifiers,” maintaining transmission chains that extend beyond their walls. The review highlights three essential axes: inequality in scientific production, predominance of a biomedical approach, and institutional fragmentation. Overcoming these limitations requires coordinated research, prison policies that guarantee therapeutic continuity, and an approach based on human rights and social justice.

In other regions of the world, such as Eastern Europe and Central Asia, up to one-third of MDR-TB cases are linked to prisons, demonstrating that this is a structural and global phenomenon [2,37]. In southern Africa, studies in South Africa show that overcrowding and treatment discontinuity continue to drive transmission despite diagnostic advances [38,39]. In Asia, the Philippines reports incidences exceeding 2400 cases per 100,000 inmates, with rising resistance and inadequate follow-up [40,41].

Taken together, the findings show that controlling multidrug-resistant tuberculosis in Latin American prisons requires overcoming three structural limitations: the scarcity of systematic evidence, institutional fragmentation, and weak attention to social determinants. Addressing these challenges demands an intersectoral approach integrating health, justice, and human rights, promoting more humane and sustainable prison policies. This orientation aligns with the World Health Organization’s End TB Strategy, which aims to reduce the global incidence of tuberculosis by 90% and associated mortality by 95% by 2035, through an approach based on equity and social protection [42]. It also resonates with the United Nations’ Sustainable Development Goals, particularly SDG 3, aimed at ensuring healthy lives and promoting well-being for all, and SDG 16, which seeks to build effective, accountable, and inclusive institutions at all levels [43]. Addressing drug-resistant tuberculosis in penitentiary contexts, therefore, is not merely a health objective but an essential ethical and social commitment to reducing inequalities and advancing towards fairer, safer, and healthier societies in Latin America and the Caribbean.

## 5. Conclusions

Drug-resistant tuberculosis in penitentiary centres across Latin America and the Caribbean represents a major public health challenge, with rates far higher than those observed in the general population and a direct impact on community transmission. The analysis reveals that scientific production in this field remains limited and uneven, concentrated in only a few countries whilst leaving significant gaps in others, which hinders a comprehensive regional understanding of the problem.

Persistent structural and programmatic shortcomings, including diagnostic delays, low coverage of drug susceptibility testing, treatment discontinuity after release, and weak coordination between prison systems and national tuberculosis programmes, further exacerbate the situation. Most studies continue to adopt a predominantly biomedical perspective, with insufficient incorporation of social determinants, prison conditions, and human rights considerations. This narrow focus limits the development of sustainable strategies capable of addressing the complexity of the issue.

Strengthening multicentre and collaborative research is essential, alongside the promotion of low-cost genomic surveillance and the generation of robust evidence to guide more comprehensive health and penitentiary policies. Future efforts must prioritise:

- Implementing systematic screening upon entry and throughout incarceration.
- Ensuring continuity of treatment after release.
- Reducing overcrowding and improving the structural conditions of detention facilities.
- Integrating social, epidemiological, and human rights approaches in the management of DR-TB in prisons.

Ultimately, controlling drug-resistant tuberculosis in prisons is not merely a health objective but an ethical and social imperative. It reflects the responsibility of States to safeguard human rights and to promote inclusive, equitable, and sustainable policies that protect both incarcerated populations and society as a whole.

## Figures and Tables

**Table 1:** Problems and gaps identified in research on drug-resistant tuberculosis among people deprived of liberty in Latin America and the Caribbean.

Identified Problem	Evidence/Knowledge Gap	References
Limited scientific production specifically on MDR-TB in prisons	Scarcity of studies in several countries; research concentrated mainly in a few contexts (Brazil, Paraguay, Peru, Colombia, Dominican Republic). Difficulty in conducting regional comparisons and designing evidence-based policies.	[6,9,13,24]
Methodological heterogeneity among studies	Differences in study designs (ecological, cohort, institutional reports). Absence of systematic drug susceptibility testing (DST) and molecular analyses in many contexts.	[5,14,30,35]
Weak integration between the prison system and national TB programmes	Lack of systematic screening upon entry, diagnostic delays, low DST coverage, and poor treatment continuity after release.	[7,16,36]
Limited consideration of social and structural determinants	Factors such as overcrowding, HIV co-infection, socioeconomic precariousness, and institutional weaknesses are mentioned but rarely addressed comprehensively.	[28,31]
Predominance of a biomedical approach	Absence of interdisciplinary perspectives integrating social, structural, and human rights dimensions in MDR-TB analysis.	[6,17]

**Table 2:** Drug-resistant tuberculosis in prisons of Latin America and the Caribbean, by region.

Region	Countries Included	Reported Incidence/ Prevalence in Prisons	Identified Structural and Social Factors	Main Challenges in DR-TB Control
**Southern Cone**	Brazil, Paraguay, Chile, Argentina, Uruguay	Documented MDR-TB outbreaks; prevalence up to 40 times higher than in the general population (Brazil, Paraguay)	Chronic overcrowding, diagnostic delays, HIV co-infection	Low coverage of drug susceptibility testing, discontinuity of treatment after release
**Andean Region**	Peru, Colombia, Ecuador	Peru: MDR and pre-XDR TB outbreaks in prisons of Lima and Callao; Colombia: documented transmission in four prisons; Ecuador: circulation of resistant lineages in Guayaquil	Deficient systematic screening, educational and socioeconomic gaps	Weak integration between prison and public health systems; lack of genomic surveillance
**Caribbean**	Cuba, Haiti, Dominican Republic	Haiti: DR-TB documented in five health institutions linked to prison populations; Dominican Republic: national survey on catastrophic costs showing high burden in prisons; Cuba: drug resistance reported between 2015–2017	Poor infrastructure, prison overcrowding, institutional weakness	Limited local scientific production, fragmented programmes, insufficient treatment continuity

## Data Availability

No new data were generated or analyzed in this study. The information supporting the findings is available in the references cited throughout the manuscript and in public access databases, such as WHO, PAHO, PubMed, Scopus, Web of Science, SciELO, and LILACS.

## Supplementary Materials

The following supporting information can be downloaded at: https://www.mdpi.com/article/10.3390/tropicalmed11040088/s1.

## Abbreviations

The following abbreviations are used in this manuscript:

DNA	Deoxyribonucleic Acid
DOT	Directly Observed Therapy
DR-TB	Drug-Resistant Tuberculosis
DST	Drug Susceptibility Testing
MDR-TB	Multidrug-Resistant Tuberculosis
PAHO	Pan American Health Organization
pre-XDR TB	Pre-Extensively Drug-Resistant Tuberculosis
SDGs	Sustainable Development Goals
TB	Tuberculosis
WHO	World Health Organization
XDR-TB	Extensively Drug-Resistant Tuberculosis
