# Supplementary material for: Drug-Resistant Tuberculosis in Prisons of Latin America and the Caribbean: A Critical Reflection on Structural Challenges and Gaps
Source: Trop Med Infect Dis. 2026 Mar 24;11(4):88. doi: 10.3390/tropicalmed11040088 (PMC13120411; doi:10.3390/tropicalmed11040088)
Supplement: Supplementary file 1 [file tropicalmed-11-00088-s001.zip › tropicalmed-4074929-supplementary.pdf]

SUPPLEMENTARY MATERIAL S1. SEARCH STRATEGY (2019–2024)

Databases searched
PubMed/MEDLINE, Scopus, Web of Science, SciELO, and LILACS.

Keywords and descriptors
“multidrug-resistant tuberculosis”, “drug-resistant tuberculosis”, “prison”, “people deprived of liberty”, “Latin America”, “Caribbean”.

Search strings (by database)

1. PubMed/MEDLINE
   ((“multidrug-resistant tuberculosis” OR “drug-resistant tuberculosis” OR MDR-TB OR DR-TB OR XDR-TB OR “rifampicin-resistant tuberculosis” OR RR-TB)
   AND (prison* OR incarcerated OR “people deprived of liberty” OR detention OR jail)
   AND (“Latin America” OR “Caribbean” OR “Latin American”))
   Filters: Publication years 2019–2024; Languages: English, Spanish.
2. Scopus
   (TITLE-ABS-KEY(“multidrug-resistant tuberculosis” OR “drug-resistant tuberculosis” OR MDR-TB OR DR-TB OR XDR-TB OR “rifampicin-resistant tuberculosis” OR RR-TB)
   AND TITLE-ABS-KEY(prison* OR incarcerated OR “people deprived of liberty” OR detention OR jail)
   AND TITLE-ABS-KEY(“Latin America” OR Caribbean OR “Latin American”))
   Filters: 2019–2024; Languages: English, Spanish.
3. Web of Science (Core Collection)
   TS=(“multidrug-resistant tuberculosis” OR “drug-resistant tuberculosis” OR MDR-TB OR DR-TB OR XDR-TB OR “rifampicin-resistant tuberculosis” OR RR-TB)
   AND TS=(prison* OR incarcerated OR “people deprived of liberty” OR detention OR jail)
   AND TS=(“Latin America” OR Caribbean OR “Latin American”)
   Refined by: Publication years 2019–2024; Languages: English, Spanish.
4. SciELO
   (“tuberculosis multirresistente” OR “tuberculosis resistente” OR “tuberculosis farmacorresistente” OR MDR-TB OR DR-TB)
   AND (prisión OR prisiones OR carcel* OR “personas privadas de libertad” OR PPL)
   AND (“América Latina” OR Caribe)
   Filters: 2019–2024; Languages: Spanish, English.
5. LILACS
   (“tuberculosis multirresistente” OR “tuberculosis resistente” OR “tuberculosis farmacorresistente” OR MDR-TB OR DR-TB)
   AND (prisión OR prisiones OR carcel* OR “personas privadas de libertad” OR PPL)
   AND (“América Latina” OR Caribe)
   Filters: 2019–2024; Languages: Spanish, English.

Additional sources

WHO and PAHO institutional reports and technical documents were reviewed to complement peer-reviewed evidence and contextualise regional surveillance and policy frameworks.
